# P02.190. Neural structural/functional and physiological correlates of massage therapy in response to physical stress

**DOI:** 10.1186/1472-6882-12-S1-P246

**Published:** 2012-06-12

**Authors:** M Zhang, M Menard, J VanMeter, L Lozier, S Browning, R Renton, A Breeden, J Brar, R Savani, P Seales, B Basdag, S Kenmore, T Mena, M Dutton, H Amri

**Affiliations:** 1 University of Maryland School of Medicine, Baltimore, USA; 2 Georgetown University Medical Center, Washington, D.C., USA; 3 University of Virginia School of Medicine, Charlottesville, USA; 4 Uniformed Services University School of Medicine, Bethesda, USA; 5 Johns Hopkins School of Medicine, Baltimore, USA; 6 Massage Therapy Clinic, Silverspring, USA

## Purpose

Massage therapy is very popular in the U.S. and rated by patients as helpful self-care modality. It is used for a variety of conditions: most commonly for chronic pain and musculoskeletal problems, but also to alleviate stress, anxiety, and depression. It has been reported that changes in regional cerebral blood flow measured by PET scanning when palm-pressure back massage was applied to subjects in prone position. However, the mechanism of massage therapy effect is not yet known. Therefore, we sought to examine the effects of massage on the acute stress response in healthy subjects using functional imaging and plasma catecholamines measurements.

## Methods

Seven healthy right-handed young males, naïve to massage therapy, qualified for the study. Exclusion criteria ranged from having depression to cardiovascular diseases. The study used a within-subject design and the cold pressor test (CPT) to trigger the acute stress response before and after a 20 minute-standardized verum or sham foot massage. Blood samples were collected at each step of the protocol. fMRI data were collected using the arterial spin labeling technique to allow for quantification of cerebral blood flow. The functional paradigm included collection of baseline (rest) and CPT both before and after massage.

## Results

There was a significant decrease in activity during stress after massage compared with pre-massage in the left caudate nucleus, rectus gyrus (Brodmann Area 11), and bilaterally in the inferior frontal gyrus. A decrease in the epinephrine and norepinephrine levels was also observed after the massage session.

## Conclusion

The decreased response following massage suggests a calming of the limbic-prefrontal cortical circuit that is activated during stress and effects on the sympathetic nervous system. These results provide preliminary evidence for the mechanism of massage therapy effects related to pain processing being related to reduction in a sympathetic response.

